# Supplementary material for: Novel Insight into the Photophysical Properties and 2D Supramolecular Organization of Poly(3,4-ethylenedioxythiophene)/Permodified Cyclodextrins Polyrotaxanes at the Air–Water Interface
Source: Materials (Basel). 2023 Jun 30;16(13):4757. doi: 10.3390/ma16134757 (PMC10342996; doi:10.3390/ma16134757)
Supplement: Supplementary file 1 [file materials-16-04757-s001.zip › materials-2468496-supplementary.pdf]

Supplementary Material

Novel insight into the photophysical properties and 2D supramolecular organization of poly(3,4-ethylenedioxythiophene)/permodified cyclodextrins polyrotaxanes at the air–water interface

Alae El Haitami[1], Ana-Maria Resmerita[2], Elena Laura Ursu[2], Mihai Asandulesa[2], Sophie Cantin[1] and Aurica Farcas[2,*]

[1] LPPI, CY Cergy Paris Université F95000 Cergy, France

[2] "Petru Poni" Institute of Macromolecular Chemistry, Romanian Academy, Grigore Ghica Voda Alley, 41A, 700487 Iasi, Romania

* Correspondence: afarcas@icmpp.ro; Tel.: +40-232-217454

S1. Characterization data of compounds

***S**1.1. ¹H-NMR spectra of Py-EDOT-Py, Py-EDOT-Py+TMe-βCD and TMe-βCD.*

The ¹H-NMR spectra evidenced the peaks of Py at 8.53 - 8.03 ppm, as well the peaks corresponding to the methylene groups from the EDOT units at 4.69 - 4.39 ppm and the peaks corresponding to the anomeric protons of TMe-βCD at 5.13 ppm and rest of the TMe-βCD protons in the interval of 3.79 - 3.20 ppm.

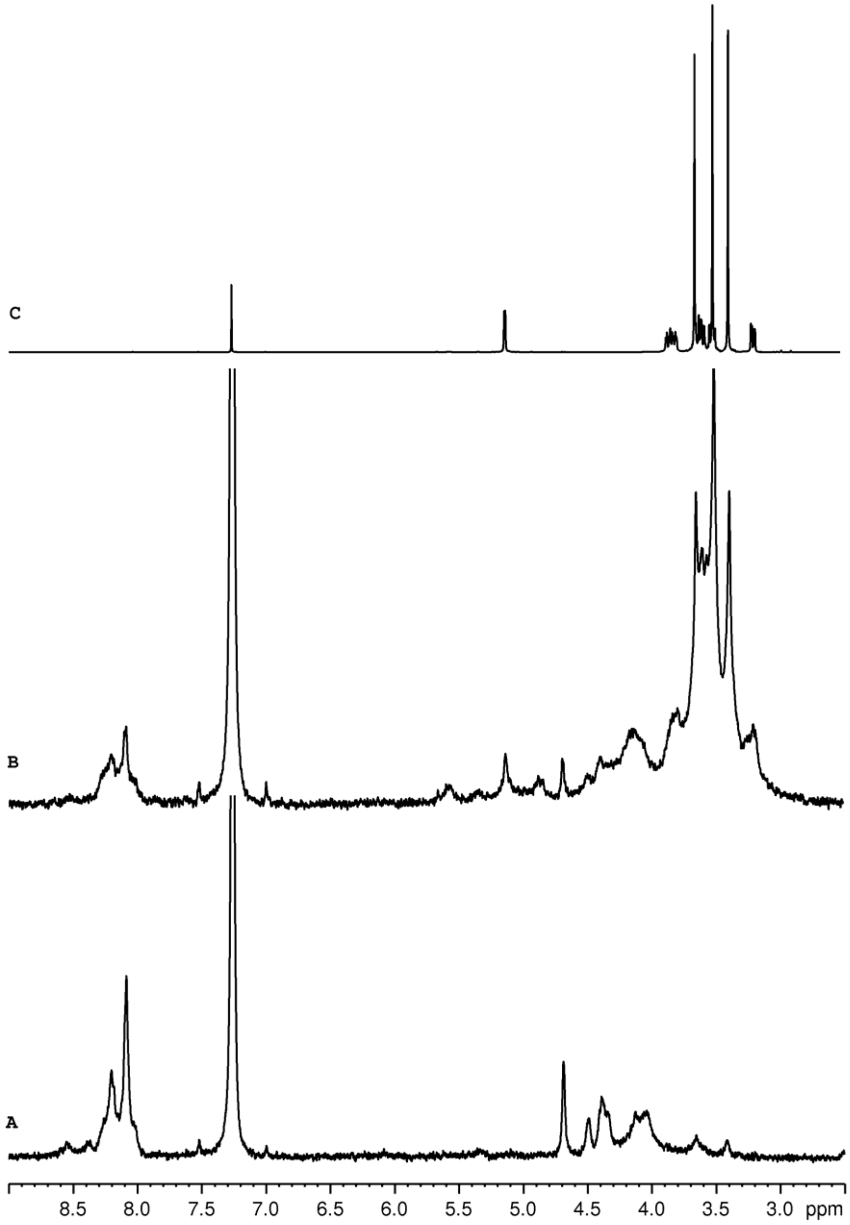

Figure S1. ¹H-NMR ($CDCl_3$, 400 MHz) spectra for: A) Py-EDOT-Py; B) Py-EDOT-Py+TMe-βCD and C) TMe-βCD.

S1.2. *MALDI MS/MS spectrum of the pristine Py-EDOT-Py+ ion species*

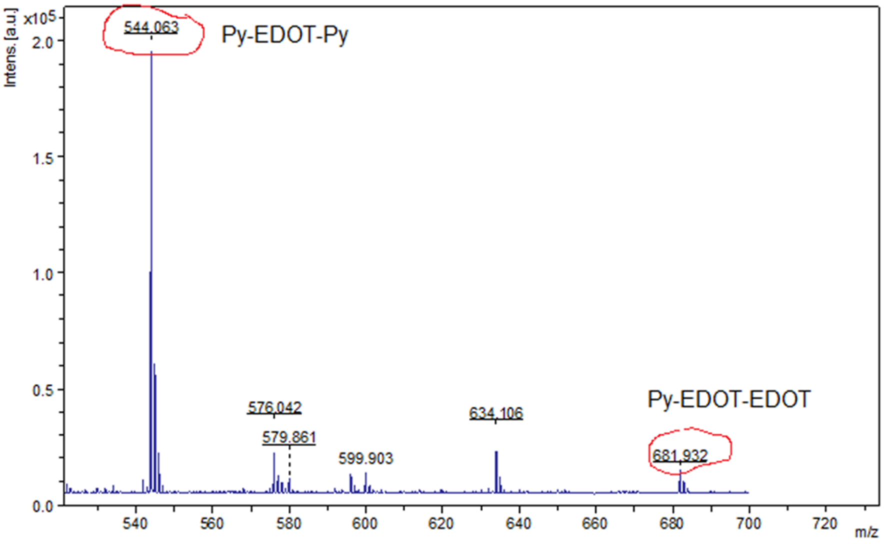

Figure S2. MALDI MS/MS spectrum of the pristine Py-EDOT-Py+ ion species.

S1.3. *FT-IR spectra of PEDOT·TMe-βCD and PEDOT·TMe-γCD*

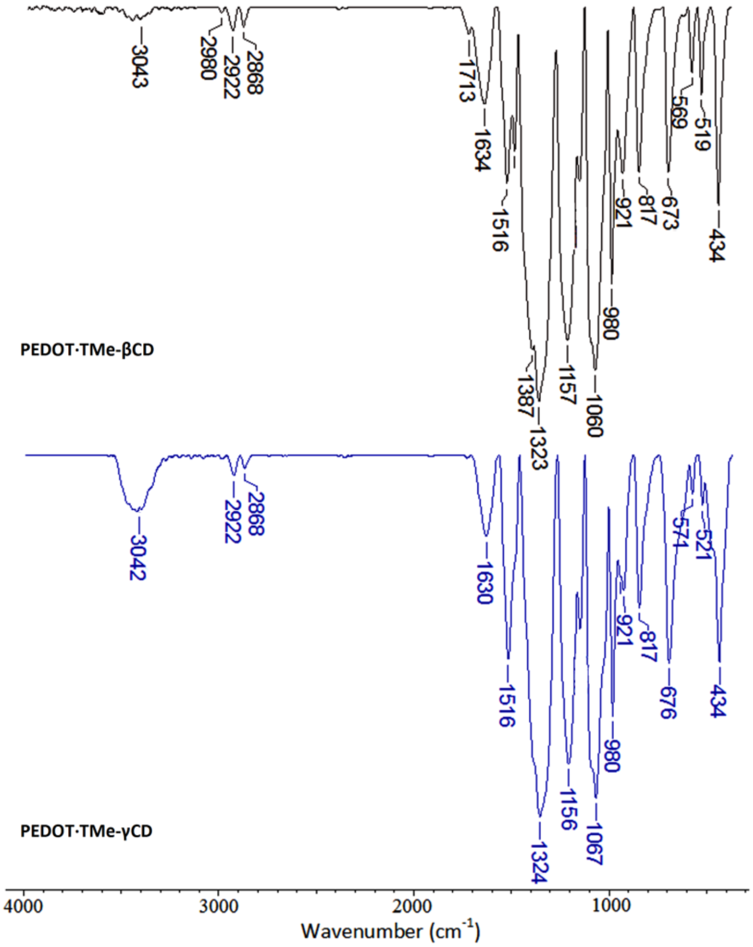

Figure S3. FT-IR spectra of PEDOT·TMe-βCD and PEDOT·TMe-γCD

S1.4. *^{1}H-NMR spectra of PEDOT·TMe-βCD and PEDOT·TMe-γCD*

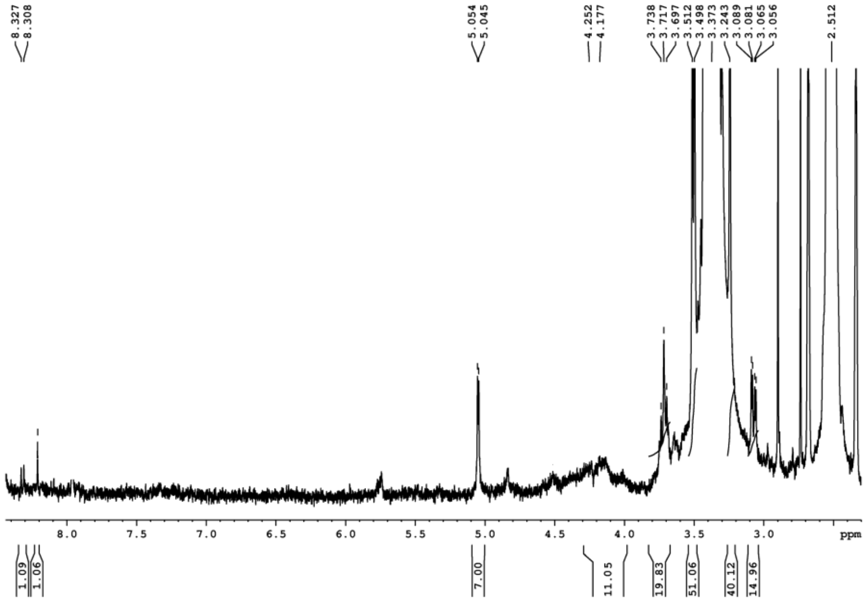

Figure S4. ^{1}H-NMR spectrum of PEDOT·TMe-βCD in DMSO-d_6.

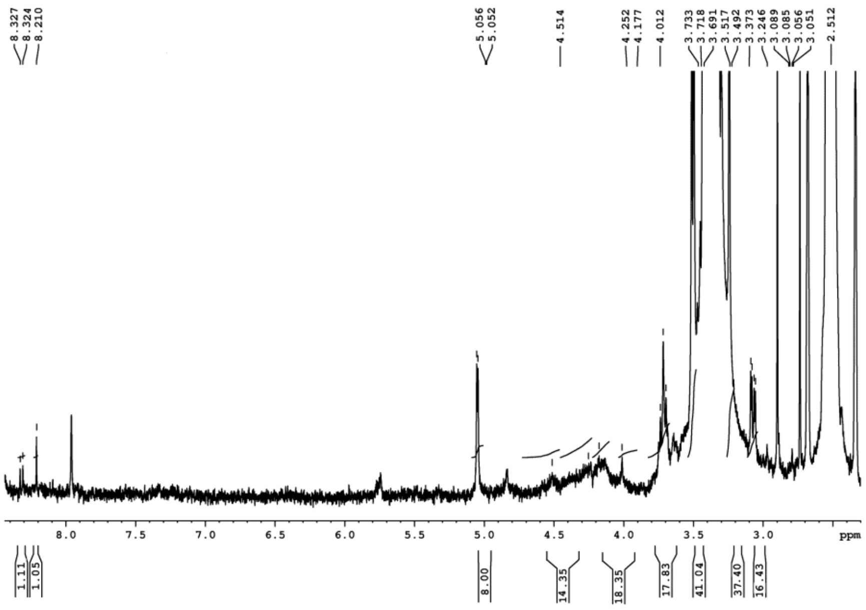

Figure S5. ^{1}H-NMR spectrum of PEDOT·TMe-γCD in DMSO-d6.

S1.5. Gel permeation chromatography (GPC)

The GPC study was performed in order to get information about the degree of TMe-βCD or TMe-γCD dethreading from the PEDOT chains under elution conditions and compared to those of the PEDOT polymer.

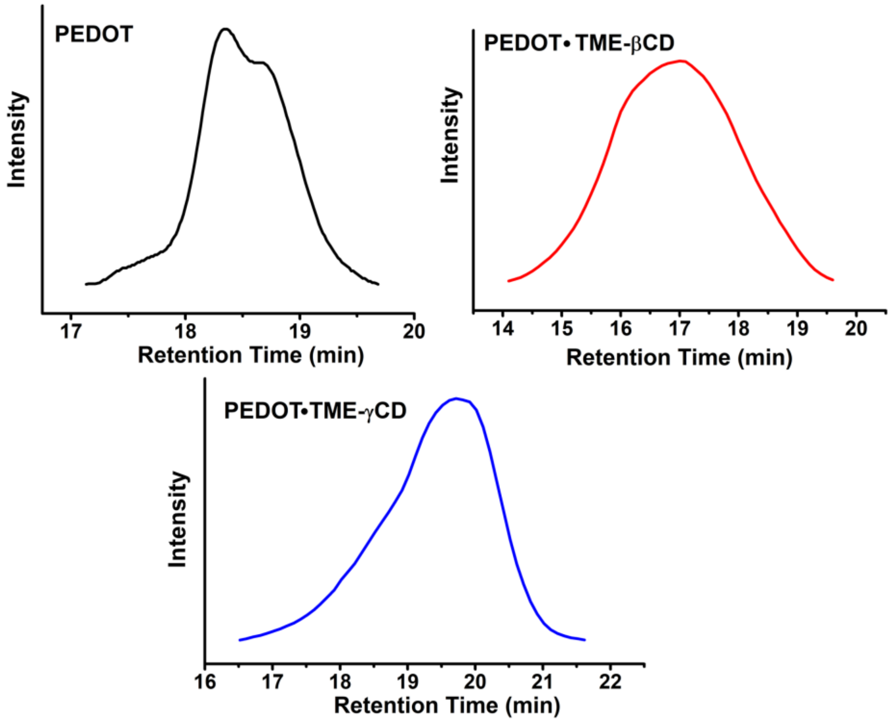

Figure S6. Comparison between the chromatograms of PEDOT, PEDOT·TMe-βCD and PEDOT·TMe-γCD.

S1.6. Thermal Analysis

Further information is provided by TGA and DSC analyses.

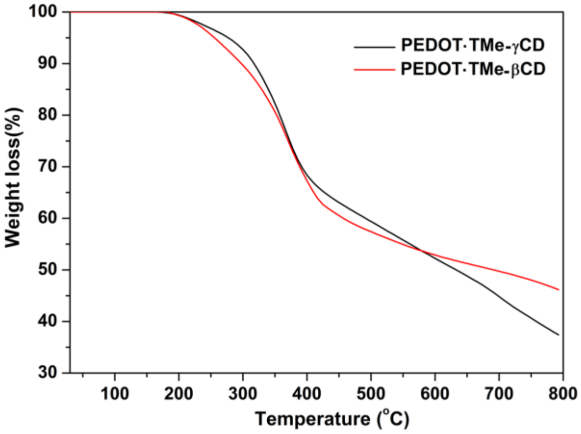

Figure S7. Thermograms of the PEDOT·TMe-γCD (black line) and PEDOT·TMe-βCD (red line).

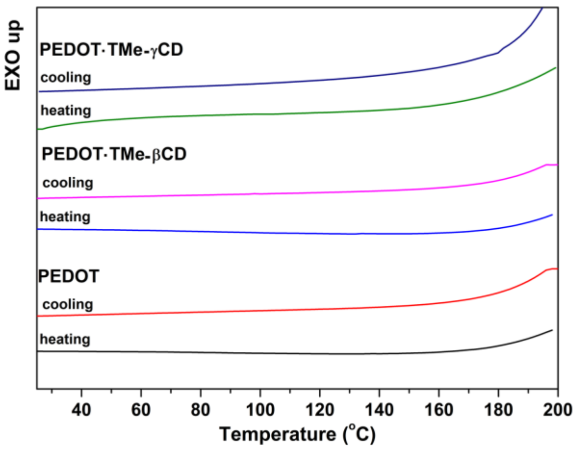

Figure S8. Second heating-cooling DSC curves of PEDOT·TMe-γCD, PEDOT·TMe-βCD and PEDOT.

S1.7. Optical properties

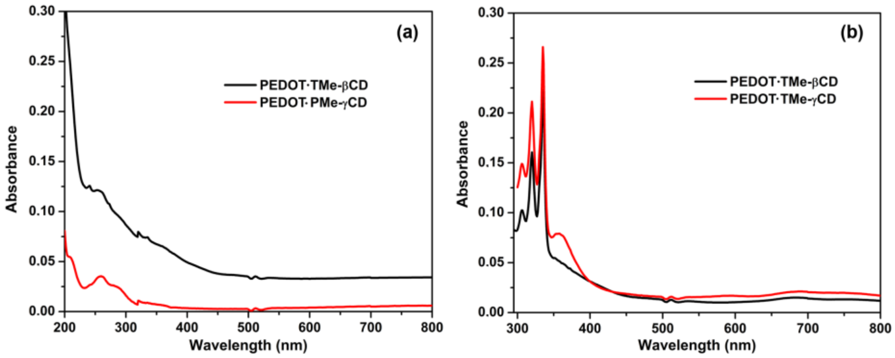

Figure S9. UV-vis absorption spectra of absorption spectra PEDOT·TMe-βCD and PEDOT·TMe-γCD in H_2O (a) and in ACN (b).

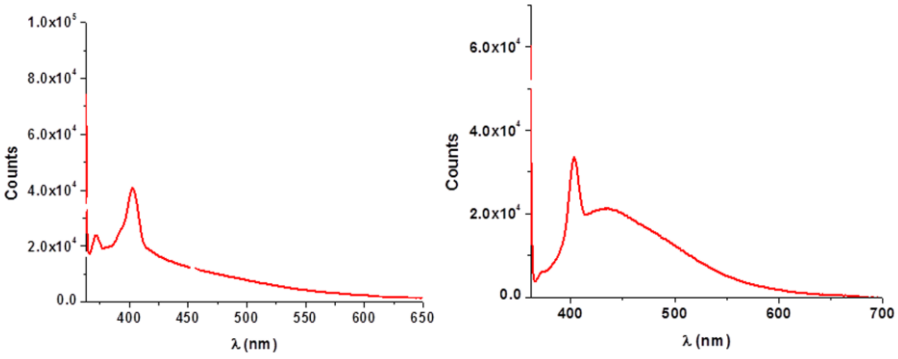

Figure S10. Fluorescence spectra of PEDOT·TMe-βCD (left) and PEDOT·TMe-γCD (right) in H_2O with λ_{ex}=355 nm.

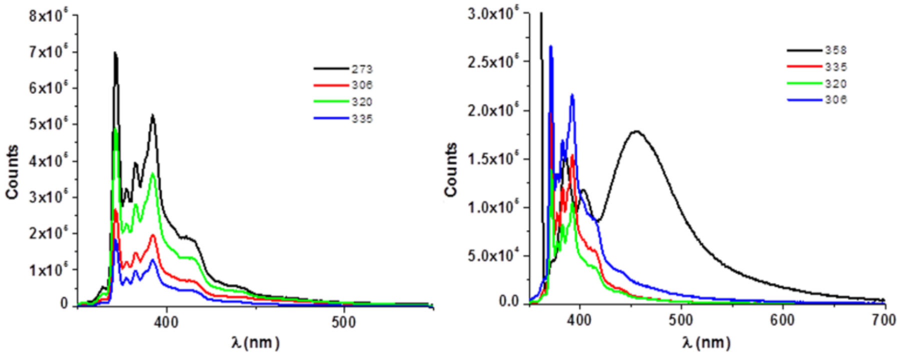

Figure S11. Fluorescence spectra of PEDOT·TMe-βCD (left) and PEDOT·TMe-γCD (right) in ACN at different excitation wavenleghts.

S1.8. Cyclic voltammograms of PEDOT·TMe-βCD and PEDOT·TMe-γCD

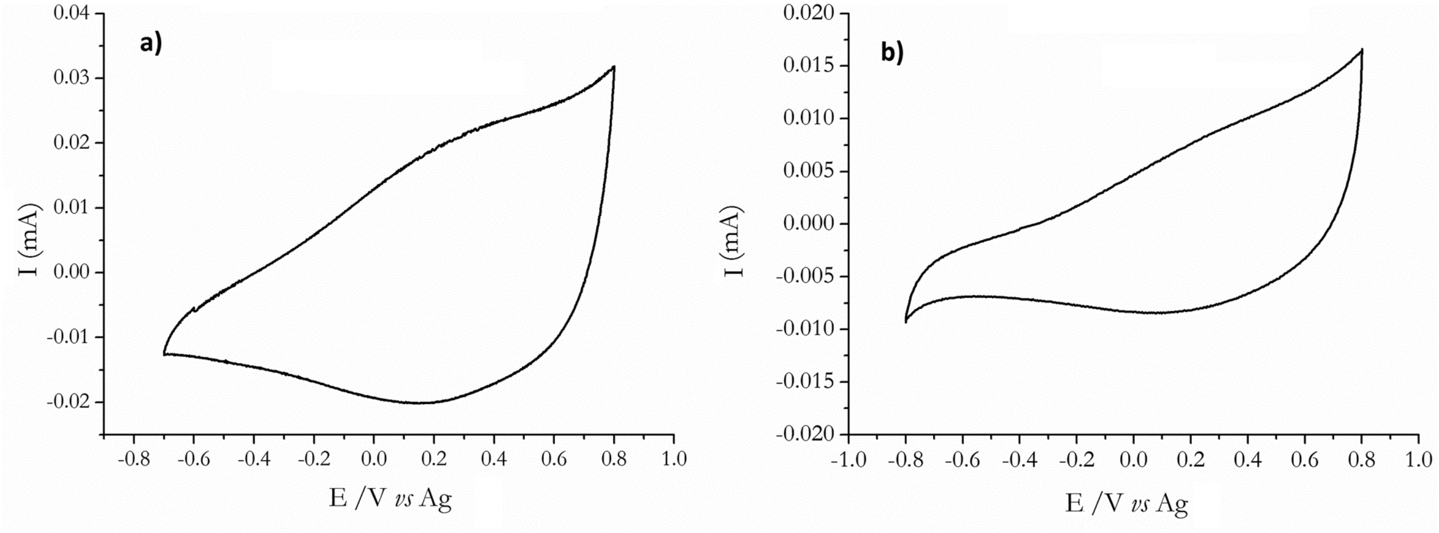

Figure S12. CV of PEDOT·TMe-βCD (a) and PEDOT·TMe-γCD (b) in 0.1 M TBAClO4)/ACN solution at scan rate 20 mV·s-1.

S1.9. The evolution of measured direct current conductivity (σ_{DC}) value against the inverse of temperature of PEDOT·TMe-βCD and PEDOT·TMe-γCD.

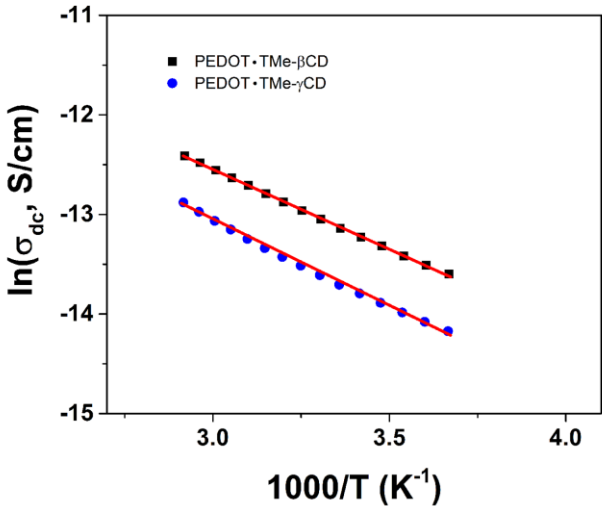

Figure S13. The presentation of σ_{DC} as a function of inverse of temperature for PEDOT·TMe-βCD and PEDOT·TMe-γCD. The linear fit functions are applied between 0 and 70 °C and represented with red lines.
